# Mortality changes after grants from the Global Fund to Fight AIDS, tuberculosis and malaria: an econometric analysis from 1995 to 2010

**DOI:** 10.1186/s12889-015-2305-1

**Published:** 2015-09-28

**Authors:** Isabel Yan, Eline Korenromp, Eran Bendavid

**Affiliations:** Department of Economics and Finance, City University of Hong Kong, Tat Chee Avenue, Kowloon, Hong Kong; Department of Public Health, Erasmus MC, University Medical Center, Postbus 2040 3000, CA Rotterdam, The Netherlands; Avenir Health, Geneva, Switzerland; Division of General Medical Disciplines, Stanford University Stanford, Stanford, CA 94305 USA; Center for Health Policy and the Center for Primary Care and Outcomes Research, Stanford University, Stanford, CA 94305 USA

**Keywords:** Global Fund, Mortality, Outcomes evaluation

## Abstract

**Background:**

Since its founding in 2002, the Global Fund to Fight AIDS, Tuberculosis, and Malaria (Global Fund) has become the dominant multilateral health financier in low- and middle-income countries. The health impact of the Global Fund remains unknown because existing evaluations measure intermediate outcomes or do not account for preexisting and counterfactual trends.

**Methods:**

We conducted an econometric analysis of data from all countries eligible to receive Global Fund grants from 1995 to 2010, prior to and during the Global Fund’s activities. We analyzed three outcomes: all-cause adult (15–59 years), all-cause under-five, and malaria-specific under-five mortality. Our main exposure was a continuous longitudinal measure of Global Fund disbursements per capita. We used panel fixed effect regressions, and analyzed mortality trends controlling for health spending, health worker density (a measure of health system capacity), gross domestic product, urbanization, and country fixed-effects.

**Results and discussion:**

We find that following Global Fund disbursements, adult mortality rate declined by 1.4 % per year faster with every $10 per capita increase in disbursements (*p* = 0.005). Similarly, malaria-specific under-five mortality declined by 6.9 % per year faster (*p* = 0.033) with every $10 high per capita Global Fund disbursements. However, we find no association between Global Fund support and all-cause under-five mortality. These findings were consistent after subanalyses by baseline HIV prevalence, adjusting for effects of concurrent health aid from other donors, and varying time lags between funding and mortality changes.

**Conclusions:**

Grants from the Global Fund are closely related to accelerated reductions in all-cause adult mortality and malaria-specific under-five mortality. However, up to 2010 the Global Fund has not measurably contributed to reducing all-cause under-five mortality.

**Electronic supplementary material:**

The online version of this article (doi:10.1186/s12889-015-2305-1) contains supplementary material, which is available to authorized users.

## Background

Development assistance to the health sector of developing countries has increased by over 3-fold between 1990 and 2012 [1, 2]. A central institution involved in this growth has been the Global Fund to Fight AIDS, Tuberculosis, and Malaria (Global Fund). From its founding in 2002 through the end of 2012, the Global Fund disbursed nearly $19 billion (current USD) in development assistance to disease control programs in nearly 140 countries [3]. During this period, the Global Fund has become the dominant multilateral funder of health programs in low- and middle-income countries.

To date, the Global Fund has focused on financing HIV/AIDS, tuberculosis, and malaria programs. Among these, HIV/AIDS programs received the most funding, and tuberculosis the least [3]. Through 2012, the Global Fund determined funding eligibility based on country income, past and ongoing Global Fund grant performance, and, for upper-middle income countries, disease burden and development assistance from other sources [4].

The Global Fund actively supports program evaluations, although these have mostly focused on process measures rather than health outcomes [5, 6]. The Global Fund’s annual Results Report calculate number of lives saved using models that rely on the efficacy of financed goods such as antiretroviral therapy (ART) to translate service delivery outputs into mortality benefits [7]. However, this approach does not account for differences between theoretical efficacy and real-world effectiveness, or for mortality changes that are unrelated to the Global Fund’s support [8].

We provide a quantitative assessment of the relationship between Global Fund disbursements and subsequent changes in key health outcomes: all-cause adult mortality, all-cause under-five mortality, and malaria-specific under-five mortality. Quantifying the mortality changes related to Global Fund support is important for several areas of global health policy. First, understanding and optimizing the Global Fund’s health outcomes has direct implications for the health of millions: the opportunity cost of ineffectiveness is high. Second, estimating the Global Fund’s effectiveness makes an important contribution to the growing science of health aid effectiveness, and is an important input for measuring the value provided by the organization [9]. Third, additional data and analyses are needed to inform the broad policy questions over the extent to which health aid improves health outcomes. Finally, the Global Fund regularly implements changes to its resource allocation decisions to maximize impact, and understanding the Global Fund’s overall relationship with mortality patterns, especially child mortality, where the Global Fund’s impact is less certain, could provide important information for future strategic planning [10–12].

## Methods

### Approach

Our overall approach examines the relationship between Global Fund disbursement and longitudinal mortality trends among all countries ever eligible to receive Global Fund grants prior to and during the Global Fund’s period of activity, from 1995 to 2010. The baseline period between 1995 and 2002 (prior to the Global Fund) allows us to analyze the role of pre-existing and baseline trends.

### Global fund support

We analyzed data from all countries that ever received Global Fund disbursements, and countries that were eligible to apply for grants but did not receive any disbursements, between 2002 and 2010. Eligible non-recipients included several countries who had applied unsuccessfully, or whose eligibility had changed over time. In compiling support, we examined grant disbursements to individual countries as well as through multi-country grants. We allocated multi-country grants evenly among recipients [8, 13].

We operationalized Global Fund support (the principal exposure) as a continuous variable of Global Fund support per capita. Global Fund support was calculated as the total disbursements between 2002 and 2010, including multi-country grants. When examining all-cause adult and under-five mortality, we used Global Fund disbursements across all disease programs (HIV, TB, and malaria); however, we used only malaria grants when examining malaria-specific mortality. We denominated Global Fund disbursements by the population size as a measure of exposure because our intent was to look at the relationship of Global Fund disbursements with population-level changes in health outcomes. In addition, our assumptions about possible mechanisms of effectiveness flowed from disbursements to health through both treatment and prevention efforts, thereby rendering the entire population as potential beneficiaries.

To allow for the time elapsed from donor disbursements, in-country recipient expenditures on disease control programs, improved delivery of health services to the intended population, and mortality effects, our models lagged the earliest possible effect between disbursements and mortality by one year; we also capped the duration through which mortality effects could be detected at three years after the earliest year of putative effect [14, 15]. For example, our models measured any mortality effects related to disbursements in 2002 over the period between 2003 and 2005.

### Mortality outcomes

We focused on three mortality indicators: all-cause adult mortality, all-cause under-five mortality, and malaria-specific under-five mortality [16–18]. We included malaria-specific under-five mortality because it is a key impact indicator for the Global Fund, the disbursements devoted to malaria have increased substantially during the study period, and malaria makes up a sizable proportion of all-cause under-five deaths in many of the countries that receive Global Fund malaria grants [8, 19]. We used annual country-level mortality estimates produced by the Institute for Health Metrics and Evaluation (IHME) [16–18, 20]. Adult mortality was measured as the probability that a 15-years-old person would die by age 59 (45q15); under-five mortality as the probability of dying before age 5 per 1000 live births (5q0); and malaria-specific under-five mortality as the probability of death from malaria per 1000 child-years at risk. We log-transformed all mortality rates to reduce skewness and because it facilitates an intuitive interpretation of changes in mortality as percentages. IHME estimates were used because they represent the most complete source of longitudinal mortality data in all countries of interest. The information used by IHME to generate its mortality estimates varies by country, but it does not include Global Fund (or any health aid) information in generating its estimates.

### Covariates

We chose and specified four time-varying covariates to examine and adjust for important potential confounders of the relationship between Global Fund disbursements and mortality changes. First, we explicitly considered the possibility that the Global Fund targeted countries with relatively more capable health systems, so that improving outcomes may appear to be related to Global Fund support whereas in reality they reflect these countries’ stronger underlying health system. We adjusted for health workforce density according to  the Global Health Workforce Statistics database, defined as the number of physicians and nurses per 100,000 population [21, 22]. This indicator is closely related to measures of health system performance such as vaccination coverage and possibly reflects the capacity of recipient countries to effectively implement public health programs. Missing country-year observations were replaced with country-specific mean, and a log-transformed density was used in the final analysis. We checked the sensitivity of the findings to the missing data by repeating the main analyses after dropping all missing data. This analysis did not substantively change the findings, and is not shown.

We used two covariates to represent the role of social and economic development as correlates of mortality changes: real gross domestic product per capita (GDPpc) measured in constant 2005 international dollars, and the percentage of population living in urban areas from the World Development Indicators [23]. Finally, we included health spending per capita [24]. Health spending may itself drive mortality reductions, and in addition may influence funding preferences: the Global Fund views health funding from recipient public sources (“Domestic counterpart Funding”) favorably, and health expenditures are an indicator of preferential Global Fund support [25]. In addition to the time-varying covariates, all analyses included country fixed-effects to control for time-invariant differences among countries, including fixed baseline or pre-existing differences. For example, if the Global Fund made early commitments based on recipients’ *baseline* health system capacity in 2002, that time-invariant difference is controlled for with country fixed effects.

### Statistical approach and sensitivity analyses

We used panel fixed effect regressions that examine within-country changes in mortality in relation to timing and levels of Global Fund disbursements. In the primary analysis, disbursements per-capita were interacted with a time trend variable. The coefficient thus represents the magnitude of the change from pre-existing mortality trends with each increase of $1 per capita in Global Fund disbursements. All models included country fixed effects and the time-varying covariates. In addition, the health workforce density covariate was included as an interaction with the main effect parameters to examine for effect modification due to the Global Fund’s possible funding selection based on health system capacity. The primary regression models, then, can be represented as follows:

$$ \begin{array}{l} Mortalit{y}_{it}={\gamma}_i+{\beta}_1\times tren{d}_t\\ {}+{\beta}_2\times tren{d}_t\times FundPerCa{p}_{it}\\ {}+{\beta}_3\times tren{d}_t\times FundPerCa{p}_{it}\times Health\; Workforce\; Densit{y}_{it}+{\boldsymbol{Z}}_{it}\boldsymbol{\alpha} \kern0.5em +{\boldsymbol{X}}_{it}\boldsymbol{\delta} +{\varepsilon}_{it}\end{array} $$

Where *Mortality*_*it*_ is the log-transformed mortality rates for country *i* in year *t*, *trend*_*t*_ is a time trend taking on the value *t* in the t^th^ year, *FundPerCap*_*it*_ are the per-capita disbursements from the Global Fund, *HWD*_*it*_ is the health workforce density, ***X***_***it***_ is a vector of country-year covariates including real gross domestic product per capita, health expenditures per capita and the percentage of population living in the urban area., and *γ*_*i*_ denotes the fixed effect coefficient of country i. The terms that explicitly relate to the mortality trend are shown in the equation; the variables that are not interacted with *trend*_*t*_ are consolidated into the matrix term ***Z***_*it*_***α***. Robust standard errors clustered by country were calculated in all analyses. Additional regression details are in Additional file 1: S1 (Statistical Models).

Several additional analyses supported the main findings. First, we repeated the primary models in two sub-analyses: one in country subsets divided into tertiles by HIV prevalence around the start of the Global Fund, and a second restricted to the subset of sub-Saharan African countries (Additional file 1: S2), because of the Global Fund’s heavy investments and the unique disease burden of HIV, tuberculosis, and malaria in this region. Second, we examined the possibility that concurrent funding from the US President’s Emergency Plan for AIDS Relief (PEPFAR) confounded the observed Global Fund effects. A variable identifying PEPFAR support was introduced alone and in interaction with the Global Fund disbursement variables to test for effect modifications (Additional file 1: S3). Third, we explored additional models, including controlling for health aid disbursements from organizations other than the Global Fund, added unadjusted models without covariates that may be influenced by Global Fund support, trimmed the most uncertain estimates, replaced time trends with year fixed effects, and added country-specific time trends (Additional file 1: S4). Fourth, we varied the lag length between disbursements and possible start of mortality effects – 1 year in the default analysis – to 2–4 years (Additional file 1: S5). Fifth, in assessing malaria mortality, we denominated disbursements per person at risk of malaria in each country (Additional file 1: S6), as alternative to per capita in the default analysis. As a final test of uncertainty, we used the mortality bounds provided by IHME to sample from the distribution of plausible values and repeated the analyses 1000 times to generate a distribution of estimated coefficients (Additional file 1: S7).

All analyses used Stata 13.1 (Statacorp Inc). All data sources are indicated in the paper. In addition, the analytic code is provided along with the article as Supporting Information files. Additional file 2 Additional file 3 and Additional file 4.

#### Ethics

This analysis has been assessed as exempt from ethics review by Stanford’s Human Subjects Research & IRB Office.

## Results

### Baseline and descriptive findings

**Fig. 1:**
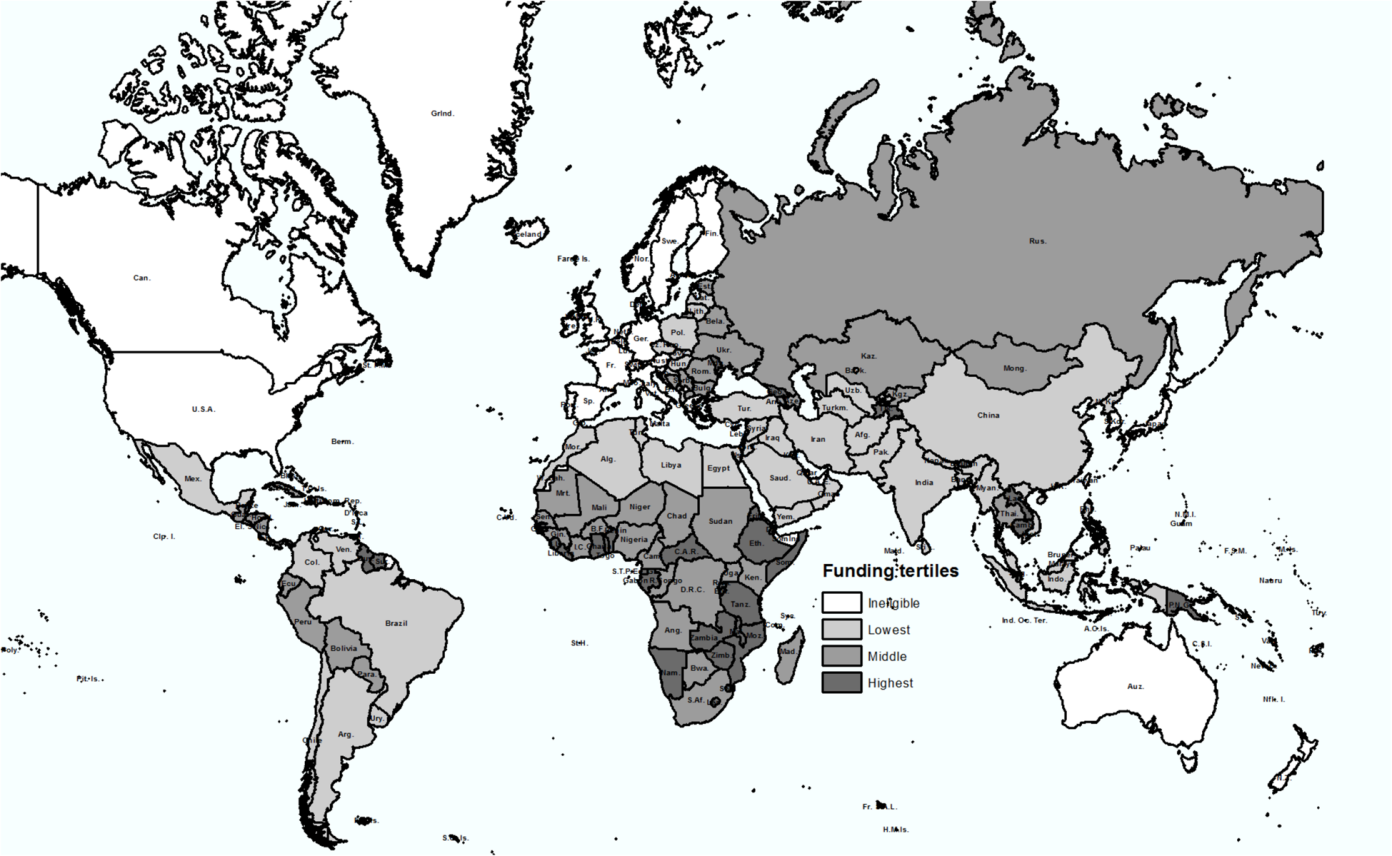
Geographical distribution of Global Fund support. Countries are shaded according to the tertile of per capita total Global Fund disbursements between 2002 and 2010, from lowest (lightest) to highest (darkest)

We analyzed data from all countries that were eligible for support from the Global Fund up to 2010 (Fig. 1). The average total disbursements per-capita between 2002 and 2010 for all study countries was $1.08 (in constant 2005 US dollars); in a wide distribution ranging from $0.1 per capita among the lowest tertile of recipients to $2.68 among the highest tertile of recipients (Table 1). The Global Fund’s per-capita disbursements increased three-fold during the period from 2007 to 2010, compared with its first five years of operation, from $0.57 to $1.73 Malaria funding also showed high variability when measured in disbursements per capita. Of the 51 countries in the highest tertile of per capita malaria disbursements, 28 were in sub-Saharan Africa. In 2002, average all-cause mortality among children and adults were 3-fold and 2-fold higher, respectively, in the highest compared with the lowest tertile countries.

**Table 1:** Descriptive characteristics of countries, at different levels Global Fund per capita disbursements

	All Eligible counties	Highest tertile	Middle tertile	Lowest tertile	% imputed data
Number of countries	152	51	51	50	--
Average real GDP per capita in 2002–2010 (in constant 2005 international dollar)	6426	3720	5174	10,406	0 %
Average public health expenditure per capita (net of GF) in 2002–2010 (in constant 2005 US dollars)	216	118	145	349	0.23 %
Average percentage of population living in urban area in 2002-2010	48	37	49	58	0 %
Total Global Fund disbursements per capita (in constant 2005 US dollars)	1.08	2.68	0.71	0.10	0 %
2002-2006	0.57	1.40	0.38	0.05	
2007-2010	1.73	4.30	1.12	0.16	
All-cause adult mortality rate^2^					
1995	246	316	254	171	0 %
2002	247	331	259	156	0 %
2007	241	326	253	150	0 %
2008	238	323	250	147	0 %
2009	236	321	248	144	0 %
2010	235	318	245	144	0 %
All-cause under-five mortality rate^3^					
1995	77	104	86	41	0 %
2002	62	86	70	33	0 %
2007	54	74	61	27	0 %
2008	52	73	60	26	0 %
2009	51	72	58	25	0 %
2010	50	71	57	25	0 %

Notes:

^1^Funding groupings used Global Fund disbursements for all sectors (HIV/AIDS, TB, malaria and Health Systems Strengthening)

^2^Adult mortality is expressed as the probability that a 15-year-old person would die by age 59 per 1000 adults (45q15)

^3^Child mortality is expressed as the number of deaths before age 5 per 1000 live births (5q0)

### Global fund and all-cause adult mortality

**Fig. 2:**
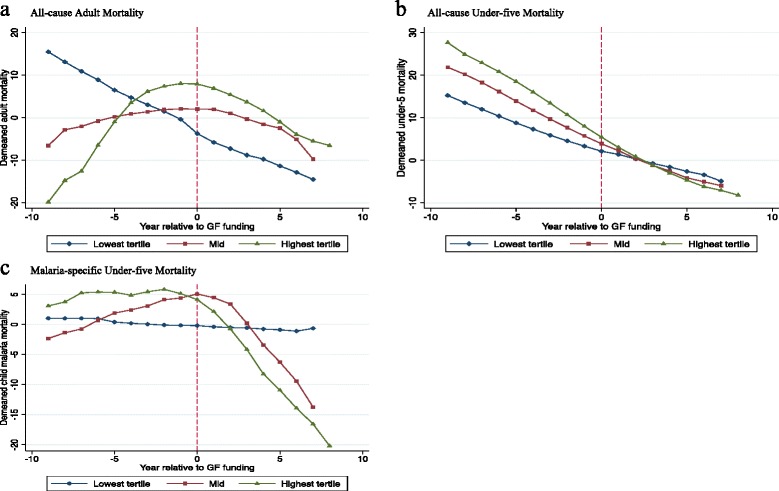
Mortality trends relative to year of first Global Fund disbursements among eligible countries, divided into tertiles of per capita disbursements: **a** all-cause adult mortality, **b** all-cause under-five mortality, and **c** malaria-specific under-five mortality. The mortality rate of each country is presented as the difference between the country’s year-specific mortality and its mean mortality between 1995 and 2010 (de-meaned). Each funding group’s mortality rate is the average of the de-meaned values for all countries within the group, thus removing from the graphs the large mortality level differences between groups that persisted throughout the time period evaluated (see Table 1), and approximating the fixed-effects regression. For each country, Year 0 represents the first year of support from the Global Fund

Higher Global Fund disbursements per capita were followed by greater declines in adult mortality. Figure 2a shows the temporal pattern of adult mortality by tertile of Global Fund support. The Figure suggests that, prior to the Global Fund, adult mortality was rising among the countries that ended up as the highest recipients, that this rise slowed just prior to start of Global Fund disbursements, and that it changed into a decline following the initiation of support. A similar pattern, though less dramatic, is seen among countries in the middle tertile: rising adult mortality rates followed by a reversal of mortality rates following start of Global Fund disbursements. In contrast, countries at the lowest tertile of support experienced declining mortality before Global Fund disbursements, and these declines continued along a similar trend after start of Global Fund support.

The regression results shown in Table 2 and Additional file 1: S2-S5 provide additional support for these relationships. We estimate that a $10 increase in Global Fund disbursements per capita was followed by a 1.4 % greater annual decline in all-cause adult mortality (*p* = 0.005). This effect was not significantly modified by an interaction with health workforce density, suggesting similar Global Fund effects on adult mortality irrespective of baseline or evolving national health system capacity. For example, in a country receiving Global Fund disbursements of $10 per capita per year, and where all-cause adult mortality declined from 400 to 345 per 1000 adults over a 5-year period (a 3 % annual decline), our analysis suggests that, absent the Global Fund, adult mortality would have dropped only from 400 to 369 per 1000 adults (a 1.6 % annual decline). In a country like South Africa with approximately 28 million adults between 15 and 60 years old, these comparative mortality trends imply approximately 190,588 fewer adult deaths associated with 5 years of Global Fund support.

**Table 2:** Relationship of Global Fund support to adult and under-five mortality changes

	All-cause adult mortality	All-cause under-five mortality	Malaria-specific child mortality
	(p-value)^5^	(p-value)^5^	(p-value)^5,6^
Global Fund $ per capita^1^	−0.0014 (0.005)	−0.0005 (0.33)	−0.0069 (0.033)
Global Fund $ per capita × HWD^2^	0.0013 (0.72)	−0.0005 (0.25)	0.0016 (0.40)
Health workforce density (HWD) ^2^	−0.0021 (0.090)	−0.0022 (0.11)	−0.0094 (0.15)
GDPpc (logged, in 2005 USD, PPP adjusted)^3^	−0.0587 (0.16)	−0.1484 (0.067)	−0.6625 (0.032)
% of urban population	0.0010 (0.77)	−0.0050 (0.23)	0.0178 (0.54)
Health expenditure per capita (logged, in 2005 USD)^4^	−0.0018 (0.92)	−0.0078 (0.60)	−0.0503 (0.55)
Number of countries	147	147	55
Number of country-year observations	2322	2322	856

Notes:

^1^Global Fund disbursements per capita in constant 2005 USD. A significant negative coefficient indicates effectiveness of Global Fund in bending down the mortality trend. The coefficient indicates that the mortality rate declined by *coefficient* × 100 percent faster per year for every $1 increase in per capita disbursements

^2^HWD is health workforce density, defined as the number of doctors and nurses per 100,000 population, an indicator of health system capacity. All HWD estimates were log-transformed. The interactions between HWD and the Global Fund variables indicate whether, at any level of Global Fund exposure, the observed outcomes changed based on HWD. The coefficient of the interactive term measures how the health workforce density in a country modifies the effectiveness of Global Fund in changing the mortality trend. There is no evidence that the effect of Global Fund on mortality was meaningfully different based on health system capacity as measured through HWD

^3^Gross domestic product per capita, log-transformed, in 2005 USD, adjusted for purchasing power parity

^4^Total health expenditures minus Global Fund disbursements from all sources per capita in 2005 USD

^5^Mortality rates are log-transformed, so that the coefficient values can be interpreted as the additional annual proportional change in mortality with each additional year of support from the Global Fund (see numerical examples in Results text)

^6^The malaria-specific models analyze countries with high, severe or extreme malaria burden. According to the Global Fund’s eligibility criteria, these are the countries with burden defined as high, severe, or extreme; countries with low or moderate burden were excluded, resulting in the analysis of 55 countries. Only malaria grants were used to examine malaria-specific mortality

### All-cause under-five mortality

Unlike for all-cause adult mortality, our analysis fails to indicate that any reductions in all-cause under-five mortality during the past decade could be attributed to Global Fund support. While the decline in under-five mortality was fastest in the countries receiving most assistance, there is no evidence of a subsequent acceleration in this trend after start of Global Fund disbursements (Fig. 2b). The regression analyses reflect these observations: changes in under-five mortality that may be attributable to the Global Fund are negligible and not statistically significant (Table 2). Similar to our findings with adult mortality, health workforce density did not significantly interact with the effect of Global Fund support. These findings make the possibility of spurious associations due to funding prioritization according to health system or program implementation capacity less likely.

### Malaria-specific under-five mortality

We find evidence accelerating malaria-specific under-five mortality declines following Global Fund malaria disbursements. Figure 2c shows that a downward bend in malaria mortality trends among the top two tertiles of recipient countries started 1–2 years following initial Global Fund malaria disbursements. The regression analysis supports this impression: each $10 increase in Global Fund disbursements per capita for malaria was associated with a 6.9 % subsequent greater annual decline in malaria-specific under-five mortality. These findings were robust when Global Fund support was expressed per person living in areas at risk of malaria (Additional file 1: S5), instead of per capita of total national population in the default analysis. When restricting the analysis to sub-Saharan Africa, the magnitude of the malaria-specific mortality declines following Global Fund disbursements is lower (3.6 %) and not statistically significant (*p* = 0.247).

### Sensitivity analyses

The direction and size of observed associations were consistent across all-cause adult and all-cause child mortality specifications when repeating the analysis in the subset of Sub-Saharan countries only. When considering the findings in relation to countries’ relative HIV burden, the declines in adult mortality were greatest in those countries with the highest baseline HIV prevalence, suggesting that reductions in HIV-related mortality have likely been instrumental in causing mortality declines (Additional file 1: S2).

We also examined the sensitivity of findings to possible synergy and/or confounding of Global Fund funding with PEPFAR funding, which clustered in an overlapping set of highest-recipient countries (Additional file 1: S3). We find that PEPFAR did not meaningfully modify the effect of the Global Fund for either adults or children. While past studies suggest that PEPFAR played a role in declining adult mortality, our findings suggest that this effect is independent from that of Global Fund support [26]. A final robustness check varied the lag duration between disbursement and start of possible mortality effects from 1 year (in the default analysis) up to 4 years (Additional file 1: S5). Extending the minimum lag between disbursements and mortality changes decreased the magnitude of the attributable reductions in malaria-specific under-five mortality.

## Discussion

Our analysis on the relationship between grants from the Global Fund and mortality changes in recipient countries suggests that Global Fund disbursements were followed by accelerated reductions in all-cause adult mortality, and these mortality reductions increased with increasing Global Fund support. This relationship was most strongly observed in countries with a high HIV prevalence, consistent with the notion that large global health initiatives have played a role in the changing epidemiology of HIV-related and, thereby, all-cause adult mortality. We also find a close relationship between malaria funding and accelerating declines in malaria-specific under-five mortality. However, we fail to find evidence that the Global Fund was associated with accelerated reductions in all-cause under-five mortality.

The lack of Global Fund effect on all-cause under-five mortality (despite large apparent effects on malaria-specific under-five mortality) requires further discussion. We observe an absence of change in child mortality trends following Global Fund grants. We speculate that Global Fund grants may have failed to contribute to accelerating child mortality declines because in many recipient countries the main causes of under-five mortality are diarrhea, pneumonia, and malnutrition (and not malaria, tuberculosis, or HIV), which Global Fund-supported disease programs do not target [27, 28]. The absence of observed changes in all-cause under-5 mortality and declines in malaria-specific under-5 mortality may result from the use of different exposures as well as from the fact that malaria makes up a small portion of all-cause under-5 mortality in many countries. We also observe that the magnitude of the declines in malaria-specific under-5 mortality is smaller in sub-Saharan Africa compared with the overall sample (Additional file 1: S2). While we cannot explain this observation, we speculate that this could possibly be explained by the relatively high burden of *falciparum* malaria, relatively high transmission risk, unique vector control challenges, or less effective use of Global Fund resources in sub-Saharan Africa.

The lack of a relationship between disbursements and all-cause under-five mortality could inform ongoing Global Fund strategic decisions to expand its support beyond the three diseases, maximizing and leveraging synergies between supported disease control programs and neonatal, infant and child health strategies and systems more broadly [29]. These considerations are articulated in Global Fund documents, but under-five mortality reduction has not been a distinct strategic priority to date [12]. External Global Fund evaluations proposed approaches for exploiting synergies between malaria and integrated management of childhood illnesses as potential approaches to enhance the Global Fund’s health impact, but such cross-program approaches have not been widely adopted to date. Our results furthermore suggest a role for measuring broad health indicators such as all-cause mortality to assess the contributions of large global health initiatives to population health.

We find a significant and robust effect on all-cause adult mortality, across all countries eligible for Global Fund support, within Sub-Saharan Africa as well as in the country subgroup with the highest HIV prevalence. In addition, we find that concurrent program funding from PEPFAR does not diminish the relationships between Global Fund support and all-cause adult mortality. Our analysis thus does not find support for a hypothesized antagonistic effect of large global health initiatives, such as those postulated to result from organizations that duplicate efforts while incurring administrative overheads without adding value [30].

### Limitations

This analysis uses aggregated, smoothed mortality estimates that were generated using sophisticated demographic and epidemiological models [31]. While some studies question the data we use for all-cause under-five mortality and malaria mortality, especially among adults outside Africa, there is broader agreement on malaria-specific under-five mortality levels and trends, and the consensus around all-cause under-five mortality is growing for many countries (with the possible exception of some countries with high HIV burden) [32, 33]. In addition, the data sources underlying these mortality estimates (such as the Demographic and Health Surveys) are of high quality [16]. Our primary analyses use the point estimates generated by IHME, though the uncertainty from the underlying data-generating process may be substantial. We make an effort to address this uncertainty by trimming the least reliable observations, thereby emphasizing observations with less uncertainty (Additional file 1: S4). In addition, the data we use for adult mortality is arrived at using lagged, smoothed GDPpc estimates (among other predictor variables). The use of GDPpc as an input to our models leave open questions about our ability to adequately control for overall level of economic development. We considered other data sources for this analysis, including the United Nations Inter-agency Group for Child Mortality Estimation for under-5 mortality and the Demographic and Health Surveys (DHS). The DHS data is available for only approximately 90 countries, and less during the study period. We also preferred IHME data because it uses a consistent methodology and underlying data sources for estimating adult, child, and malaria-specific mortality, thus limiting the measurement challenges that might arise from using different estimation methods.

Our fixed effects approach relaxes some of the limitations to causal inferences from ecological associations, yet unmeasured drivers of mortality changes that may cluster with the Global Fund in time and in geographical distribution remain a concern. Notably, it is possible that receipt of Global Fund support may correlate with a country’s intrinsic public health capacity [10]. Our inclusion of health system capacity measures (proxied by health workforce density), and their possible interaction with Global Fund funding, however, fail to support this concern. Another concern is that the observed declines in adult mortality may simply reflect the dynamic ebb of the HIV epidemic that would have occurred also without Global Fund funding. While this is not a hypothesis that our analysis can fully exclude, this is less likely to explain our findings given the Global Fund’s large infusion of financial resources to ART programs, the high efficacy of ART in reducing mortality among HIV+ individuals, the dose–response relationship that we find, whereby greater Global Fund disbursements led to greater subsequent declines in mortality, and the dramatic reversal of adult mortality trends after the introduction of ART documented in other contexts [34, 35]. Strong causal inference is challenging in the absence of *ex ante* evaluation design or natural experiments, and our aim is to make a careful and measured attempt to estimate the Global Fund’s impacts despite the challenging causal landscape.

While we accounted for possible interactions between the Global Fund and PEPFAR, as well as controlled for health aid besides the Global Fund, we did not assess interactions between Global Fund and other major multi-country donors such as the US President’s Malaria Initiative [36]. Our study does not preclude additional important survival benefits from of concurrent economic development, health system improvement, and technological advancements; nevertheless, the multivariate panel regressions suggest that the Global Fund had an effect on accelerating adult mortality reductions. The observational approaches we used in this analysis demonstrate important associations of the Global Fund with mortality changes, but cannot prove a causal link. Rather, we show that the epidemiological evidence is consistent with three narratives: that the Global Fund played a role in the reduction of adult mortality, that it played a role in reducing malaria burden among children, and that it did not measurably contribute to all-cause mortality reductions among under-5 children. We do not claim to prove these narratives, but rather raise the burden of proof for any claims to the contrary. The study provides a first-order estimate of the Global Fund’s relationship with mortality changes, though the effects may be variable in smaller country subsets or individual countries.

## Conclusion

Global Fund support was significantly associated with accelerated reductions in adult mortality and malaria-specific under-five mortality, across all recipient countries and within the subsample of Sub-Saharan countries, and independent from effects of PEPFAR’s concurrent funding. However, we observe no significant association between Global Fund support and relative changes in all-cause under-five mortality. These findings are consistent with an important positive health impact achieved so far, as well as ongoing new opportunities for this global health initiative to further enhance its impact.

### Supplementary material

Refer to Web version for supplementary material.

## Additional files

Additional file 1:
**Appendix S1: **Statistical Models; **Appendix S2.** Global Fund Effect in Country Subsets; **Appendix S3.** Global Fund Interactions with PEPFAR and Health Workforce; **Appendix S4.** Additional Specifications; **Appendix S5.** Analyses Using Different Lag Durations; **Appendix S6.** Malaria Funding Exposure per Person at Risk; **Appendix S7.** Sampling From Mortality Uncertainty Bounds. (DOCX 349 kb) Additional file 2:
**Code for producing all-cause adult mortality findings.** (DO 26 kb) Additional file 3:
**Code for producing all-cause child mortality findings.** (DO 26 kb) Additional file 4:
**Code for producing under-five malaria mortality findings.** (DO 21 kb)
