# Supplementary material for: Mortality changes after grants from the Global Fund to Fight AIDS, tuberculosis and malaria: an econometric analysis from 1995 to 2010
Source: BMC Public Health. 2015 Sep 28;15:977. doi: 10.1186/s12889-015-2305-1 (PMC4587875; doi:10.1186/s12889-015-2305-1)
Supplement: Additional file 1: — Appendix S1: Statistical Models; Appendix S2. Global Fund Effect in Country Subsets; Appendix S3. Global Fund Interactions with PEPFAR and Health Workforce; Appendix S4. Additional Specifications; Appendix S5. Analyses Using Different Lag Durations; Appendix S6. Malaria Funding Exposure per Person at Risk; Appendix S7. Sampling From Mortality Uncertainty Bounds. (DOCX 349 kb) [file 12889_2015_2305_MOESM1_ESM.docx]

**Mortality Changes After Grants From the Global Fund to Fight AIDS, Tuberculosis and Malaria: An Econometric Analysis**

**Supplementary appendix**

Isabel Yan, Eline Korenromp, Eran Bendavid

This Supplementary Appendix provides greater detail on the statistical models (SA1) and the results of sensitivity and robustness analyses (SA2-SA6).

Table of Contents

Supplementary Appendix SA1: Statistical Models 2

Supplementary Appendix SA2: Global Fund Effect in Country Subsets 4

Supplementary Appendix SA3: Global Fund Interactions with PEPFAR and Health Workforce 10

Supplementary Appendix SA4: Additional Specifications 12

Supplementary Appendix SA5: Analyses Using Different Lag Durations 14

Supplementary Appendix SA6: Malaria Funding Exposure per Person at Risk 16

# Supplementary Appendix SA1: Statistical Models

Variable definitions:

| **Dependent variable** | **Description** |
| --- | --- |
| ${Mortality}_{it}$ | Log of country-level all-cause adult, all-cause under-five mortality ratio or malaria under-five mortality rate.  Adult mortality ratio was measured as the probability that a 15-years-old person would die by age 59 (45q15); under-five mortality ratio as the probability of dying before age 5 per 1,000 live births (5q0); and malaria-specific under-five mortality rate as the probability of death from malaria per 1,000 child-years at risk. |
| **Covariates** | **Description** |
| ${FundPerCap}_{it}$ | Total of all Global Fund disbursements for country i in years t-2, t-1, and t in constant 2005 US dollars divided by population size for year t. |
| ${trend}_{t}$ | A time trend variable that takes the value t in the t^th^ year. |
| ${Health Workforce Density}_{it}$ | Natural log of the health workforce density. The density is defined as the number of physicians, nurses and midwifes per 1,000 population. |
| ${RealGDPPerCap\_PPP}_{it}$ | Natural log of real gross domestic product per capita (GDPpc) at purchasing power parity measured in constant 2005 international dollars for country i in year t. |
| ${HealthExpPerCap}_{it}$ | Natural log of health expenditures in constant 2005 US dollars (net of Global Fund) per capita for country i in year t. |
| ${Urbanization}_{it}$ | Percentage of population living in the urban area of country i at year t. |

We specified the models of the mortality outcome of interest as a linear function of the Global Fund’s disbursements per capita. All analyses use the variable ${FundPerCap}_{it}$ as the Global Fund disbursement per capita to country i in the three-year window preceding year t. The specification of the regression models for the overall adult and overall child mortality is given as follows:

${Mortality}_{it}$ = $\gamma_{i}$+ $\beta_{1}\times$ ${trend}_{t}$

+ $\beta_{2}\times$ ${trend}_{t}{\times FundPerCap}_{it}$

+ $\beta_{3}\times$ ${trend}_{t}{\times FundPerCap}_{it}\times{Health Workforce Density}_{it}$ + $\boldsymbol{Z}_{it}\boldsymbol{\alpha}$ +$\boldsymbol{X}_{it}\boldsymbol{\delta}+\varepsilon_{it}$ (1)

where $\boldsymbol{X}_{it}$ is the vector of covariates which include real gross domestic product per capita, health expenditures per capita and the percentage of population living in the urban area. $\gamma_{i}$ denotes the fixed effect coefficient of country i. The terms that explicitly affect the mortality trend are shown in the equation, and the counterpart terms not interacted with ${trend}_{t}$ are consolidated into the matrix term $\boldsymbol{Z}_{it}\boldsymbol{\alpha}$.

The coefficient $\beta_{2}$ captures the effect of Global Fund on the mortality trend. A significant negative coefficient indicates effectiveness of Global Fund in bending down the mortality trend. That is, this coefficient indicates that the mortality rate declined by $\beta_{2}\times100$ percent faster per year for every $1 increase in per capita disbursements.

$\beta_{3}$ measures how the health workforce density in a country modifies the effectiveness of Global Fund in changing the mortality trend. The estimates of both $\beta_{2}$ and $\beta_{3}$ are reported in Table 2 of the main paper. If $\beta_{3}$ is significant, it indicates that the mortality rate change measured by $\beta_{2}$ is modified by $\beta_{3}$for every 100 percent increase in the health workforce density.

The specification of the under-5 malaria mortality regression is similar to equation (1) above except that the Global Fund variable is measured as the malaria-targeted Global Fund disbursement per capita.

# Supplementary Appendix SA2: Global Fund Effect in Country Subsets

SA2-1: Global Fund relationship with mortality changes in Sub-Saharan countries: This table parallels the manuscript’s main results table. In this table, however, only Sub-Saharan African countries are included. The Notes from Table 2 apply to used for this Table as well.

We note the following differences:

1. The significance of the effect size of each Global Fund $ per capita on all-cause adult mortality moved from p=0.005 to p=0.116 between the all-country analysis and sub-Saharan country analysis, and the effect size is slightly decreased.
2. Health expenditures per capita are more consistently related to decreasing mortality in the Sub-Saharan country group.
3. There is a negative and significant interaction between Global Fund disbursements and health workforce density for both all-cause adult mortality and all-cause under-five mortality. That is, at each level of disbursements, higher health workforce density is related to greater reductions in mortality. This could reflect that health system capacity constitutes a critical condition (or barrier) for effective disease control program implementation in sub-Saharan Africa.
4. The effect size of each Global Fund malaria $ per capita on malaria-specific under-5 mortality is smaller, and the p-value insignificant in comparison with the all-country group. This could result partially from loss of statistical power; in addition, it may be related to the observation that health system capacity plays an important role in enabling (or, when weak, limiting) the effect of Global Fund disbursements in Sub-Saharan Africa, combined with the high level of malaria funding. This may suggest diminishing returns to malaria investments. Indeed, when we introduce a (Global Fund $ per capita)^2 (square) term, we observe a pattern consistent with diminishing returns: a negative (and significant) coefficient on the Global Fund $ per capita term and a positive (and significant) coefficient on the Global Fund $ per capita-squared term, suggesting mortality effects at lower disbursements that is not proportionally increased with increasingly higher levels of disbursements (results not shown in Table and available from the authors).

|  | All-cause adult mortality (p-value) | All-cause under-five mortality (p-value) | Malaria-specific child mortality (p-value) |
| --- | --- | --- | --- |
| Global Fund $ per capita | -0.0009  (0.116) | 0.0002  (0.669) | -0.0031  (0.304) |
| Global Fund $ per capita$\times$HWD | **-0.0009  (0.071)** | **-0.0011**  **(0.017)** | -0.0002  (0.934) |
| Health workforce density (HWD) | 0.0010  (0.711) | **0.0057**  **(0.007)** | 0.0002  (0.941) |
| GDPpc (logged, in 2005 USD, PPP adjusted) | **0.0964**  **(0.069)** | **0.0899**  **(0.053)** | -0.1387  (0.124) |
| % of urban population | -0.0003  (0.975) | -0.0251  (0.207) | **0.0172**  **(0.096)** |
| Health expenditure per capita (logged, in 2005 USD) | **-0.0741**  **(0.001)** | -0.0251  (0.207) | **-0.2500**  **(0.000)** |
| Number of countries | 46 | 46 | 39 |
| Number of country-year observations | 727 | 727 | 615 |

Mortality trends relative to start of Global Fund disbursements among Sub-Saharan African Countries. The three panels are parallel to those in Figure 2, restricted to 46 Sub-Saharan African countries. Adult mortality patterns among the highest and middle tertiles of recipient countries were similar to the all-country set.

| Panel SA2a: All-cause Adult Mortality (Sub-Saharan African countries) | Panel SA2b: All-cause Under-five Mortality (Sub-Saharan African countries) |
| --- | --- |
|  |  |
|  |  |
| Panel SA2c: Malaria-specific Under-five Mortality (Sub-Saharan African countries) |  |

|  |
| --- |

SA2-2: Global Fund relationship with adult mortality changes in High, Middle, and Low HIV prevalence countries: Countries were grouped based on tertiles of initial HIV prevalence (average adult HIV prevalence between 2001 and 2003, prior to effective Global Fund disbursements): highest tertile (prevalence 2.2-27.6%); middle tertile (prevalence 0.5-2.1%); and lowest tertile (prevalence <0.5%). The results are shown for the continuous specification of all-cause adult mortality. These findings suggest that the strongest observed relationship between the Global Fund and subsequent adult mortality reductions took place in countries with high HIV burden. This is consistent with the proposed mechanism for adult mortality reduction through expansion of antiretroviral therapy and ensuing reductions in HIV-related mortality.

|  | **All-cause adult mortality** | | |
| --- | --- | --- | --- |
|  | **High HIV Prevalence**  **(p-value)** | **Mid HIV Prevalence**  **(p-value)** | **Low HIV Prevalence**  **(p-value)** |
| Global Fund $ per capita | **-0.0012  (0.051)** | 0.0006  (0.726) | 0.0003  (0.809) |
| Global Fund $ per capita$\times$HWD | -0.0001  (0.989) | 0.0005  (0.541) | -0.0004  (0.471) |
| Health workforce density (HWD) | 0.0003  (0.916) | 0.0001  (0.993) | 0.0014  (0.425) |
| GDPpc (logged, in 2005 USD, PPP adjusted) | 0.0604  (0.220) | -0.0636  (0.112) | **-0.1932**  **(0.001)** |
| % of urban population | -0.0020  (0.790) | -0.0006  (0.876) | 0.0002  (0.965) |
| Health expenditure per capita (logged, in 2005 USD) | **-0.0735**  **(0.001)** | 0.0271  (0.233) | 0.0349  (0.156) |
| Number of countries | 50 | 49 | 46 |
| Number of country-year observations | **-0.0012  (0.051)** | 0.0006  (0.726) | 0.0003  (0.809) |

SA2-3: Global Fund relationship with all-cause under-5 mortality in countries with low and high share of pneumonia and diarrhea as causes of child mortality. In this analysis, we divide the countries into two groups: the first group consists of countries with high burden of diarrhea and pneumonia, and the second group consists of the rest of the countries. Countries are classified as high diarrhea and pneumonia burden countries if both their death rates of diarrhea and pneumonia in the 0-14 age group are above the median of the sample countries. We then estimate the all-cause under-5 mortality regression in each sub-sample. The results are reported in the table below. The baseline regression from the main paper is replicated in the first column for comparison. The results indicate that GF funding has a larger (and nearly significant at the p=0.05 level) relationship with all-cause under-5 mortality for countries with low burden of diarrhea and pneumonia, but not for countries with high burden of these diseases. This supports the notion that countries burdened with other leading causes of death among under-5 children reaped a lower benefit from GF disbursements.

|  | All-cause under-5 mortality (p-value) | | |
| --- | --- | --- | --- |
|  | All countries  (default baseline regression) | Countries with high diarrhea and pneumonia burden in the young age group | Countries with low diarrhea and pneumonia burden in the young age group |
| Global Fund $ per capita | -0.0005  (0.330) | -0.0004  (0.475) | -0.0024  (0.067) |
| Global Fund $ per capita$\times$HWD | -0.0005  (0.250) | -0.0005  (0.269) | 0.0001  (0.879) |
| Health workforce density (HWD) | -0.0022  (0.110) | -0.0007  (0.708) | -0.0012  (0.572) |
| GDPpc (logged, in 2005 USD, PPP adjusted) | -0.1484  (0.067) | -0.1152  (0.238) | **-0.2202**  **(0.012)** |
| % of urban population | -0.0050  (0.230) | 0.0001  (0.998) | **-0.0111**  **(0.030)** |
| Health expenditure per capita (logged, in 2005 USD) | -0.0078  (0.600) | 0.0092  (0.648) | -0.0203  (0.264) |
| Number of countries | 147 | 76 | 71 |
| Country-year observations | 2322 | 1193 | 1129 |

# Supplementary Appendix SA3: Global Fund Interactions with PEPFAR and Health Workforce

This section provides the complete details of our work to examine possible interactions between mortality effects of funding from the Global Fund and complementary funding from the US President’s Emergency Plan for AIDS Relief (PEPFAR, for HIV predominantly).

The overall patterns of PEPFAR disbursements are shown above in Figure SA3-1 below.

**Figure SA3-1: Disease-Specific Global Fund and PEPFAR (HIV-targeted) disbursements between 2002-2010**


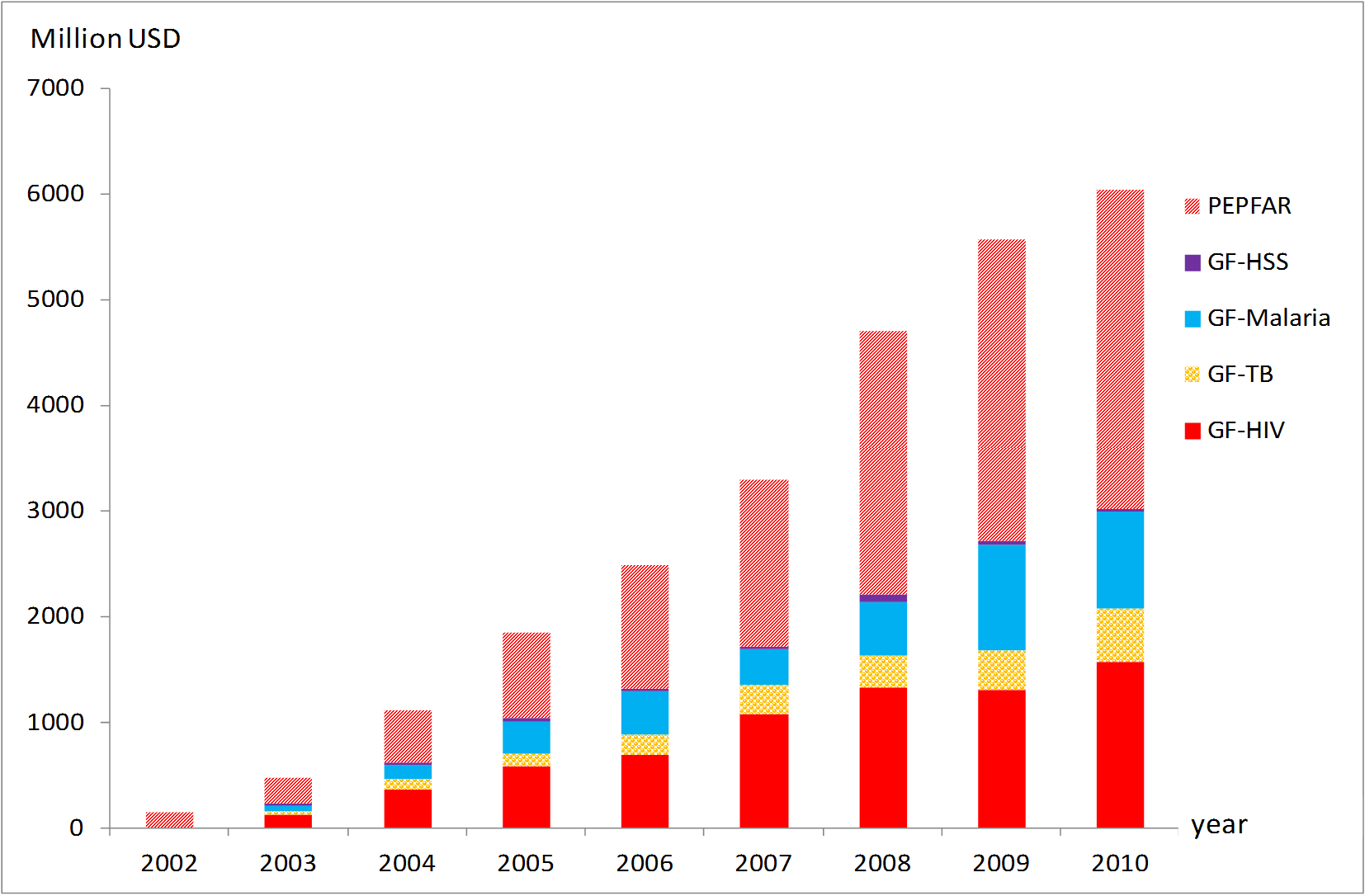


Note to Figure SA3-1: GF-HSS, GF-Malaria, GF-TB, and GF-HIV denote GF funding targeted at health system strengthening, malaria, tuberculosis, and HIV respectively. Global Fund recipient countries increased from 1 in 2002, 95 in 2003 to 132 in 2010; PEPFAR recipient countries increased from 15 in 2002-2007 to 65 in 2008 and 88 in 2010.

The purpose of this sensitivity analysis is to explore whether PEPFAR’s additional funding meaningfully modified the estimated effects of Global Fund disbursements on all-cause adult mortality. We explore this by allowing for unique mortality patterns in PEPFAR’s initial focus countries, a group of 15 countries that received 88% of PEPFAR’s financial and technical assistance. Among PEPFAR’s focus countries, 11 were also among the top tertile of Global Fund disbursement countries (Botswana, Côte d'Ivoire, Ethiopia, Guyana, Haiti, Mozambique, Namibia, Rwanda, Tanzania, Uganda, and Zambia).

The thus refined panel regression described in the following Table show a persistent Global Fund effect, without evidence for synergy or a negative interaction between the effects of PEPFAR (focus country status) and Global Fund funding.

|  | **All-cause adult mortality** | **Under-5 mortality** |
| --- | --- | --- |
| Global Fund $ per capita | **-0·0014**  **(0·000)** | -0·0004  (0·380) |
| Global Fund $ per capita$\times$PEPFAR focus country status | 0·0001  (0·350) | 0·0001  (0·207) |
| PEPFAR disbursements per capita | -0.0004  (0.71) | -0.0003  (0.79) |
| Health workforce density (HWD) | **-0·0020**  **(0·053)** | -0·0020  (0·147) |
| GDPpc (logged, in 2005 USD, PPP adjusted) | -0·0606  (0·108) | **-0·1640**  **(0·036)** |
| % of urban population | -0·0011  (0·731) | -0·0064  (0·131) |
| Health expenditure per capita (logged, in 2005 USD) | 0·0089  (0·597) | 0·0036  (0·812) |
| Number of countries | 147 | 147 |
| Number of country-year observations | 2182 | 2182 |

# Supplementary Appendix SA4: Additional Specifications

Specification SA4-1: Model adjusted for non-Global Fund health aid disbursements per capita. Estimates for non-Global Fund health aid taken from the OECD Creditor Reporting System. These non-Global Fund disbursements do not modify the Global Fund’s relationship with mortality.

|  | All-cause adult mortality (p-value) | All-cause under-five mortality (p-value) |
| --- | --- | --- |
| Global Fund $ per capita^3^ | **-0.0014  (0.010)** | -0.0006  (0.311) |
| Global Fund $ per capita$\times$HWD | 0.0001  (0.760) | -0.0004  (0.374) |
| Other non-GF $ per capita | -0.0004  (0.669) | -0.0008  (0.412) |
| Other non-GF $ per capita$\times$HWD | 0.0002  (0.623) | 0.0003  (0.507) |
| Health workforce density (HWD) | **-0.0024**  **(0.057)** | **-0.0028**  **(0.072)** |
| GDP per capita (logged, in 2005 USD, PPP adjusted) | -0.0692  (0.115) | **-0.1440**  **(0.078)** |
| % of urban population | 0.0003  (0.931) | -0.0069  (0.110) |
| Health expenditure per capita (logged, in 2005 USD) | 0.0001  (0.998) | -0.0073  (0.642) |
| Number of countries | 143 | 143 |
| Number of country-year observations | 2149 | 2149 |

Specification SA4-2: Model without adjustments for covariates that may be influenced by Global Fund disbursements. Covariates such as health workforce density and health spending may be influenced (for example, improved) by Global Fund disbursements, thus forming part of the causal pathway by which Global Fund funding ultimately impacts mortality – in which case these covariates bias the relationship under study. To see if this is the case, we conducted a panel regression that does not adjust for health workforce density and health spending. This analysis shows a persistent effect of between Global Fund supports on accelerated all-cause adult mortality decline. In addition, for all-cause under-five mortality, this ‘unadjusted’ analysis finds a possible negative (beneficial) relationship between disbursements and subsequent accelerated child mortality decline. Since the latter relationship was not evident from our default analysis, this sensitivity analysis may be consistent with some impact on under-5 mortality that is mediated by effects of Global Fund support on improving health workforce density and/or health spending.

|  | All-cause adult mortality (p-value) | All-cause under-five mortality (p-value) |
| --- | --- | --- |
| Global Fund $ per capita | **-0.0013  (0.000)** | **-0.0010**  **(0.005)** |
| Number of countries | 152 | 152 |
| Number of country-year observations | 2432 | 2432 |

Specification SA4-3: Adult and all-cause under-5 mortality after removing the most uncertain observations.

The IHME mortality data is estimated with uncertainty, and we incorporate the information contained in those uncertainty bounds to check our estimations. We take the uncertainty underlying the IHME data into account with a robustness check in which country-year observations with high underlying data uncertainty are excluded. The IHME data includes three values for the mortality estimates – best estimate, high, and low. The range of estimates is related to the quality and availability of underlying data. We then use these uncertainty bounds to remove the 5% of observations with the highest relative uncertainty measured using ((high estimate – low estimate)/best estimate) as our estimate of the relative uncertainty. The results, shown in the table below, show that the model estimates are mostly unaffected by the most uncertain estimates

|  | All-cause adult mortality (p-value) | All-cause under-five mortality (p-value) |
| --- | --- | --- |
| Global Fund $ per capita | **-0.0015  (0.004)** | -0.0002  (0.65) |
| Global Fund $ per capita$\times$HWD | 0.0001  (0.71) | -0.0002  (0.73) |
| Health workforce density (HWD) | **-0.0022**  **(0.089)** | **-0.0025**  **(0.060)** |
| GDPpc (logged, in 2005 USD, PPP adjusted) | -0.0598  (0.16) | -0.1197  (0.13) |
| % of urban population | 0.0004  (0.91) | -0.0060  (0.15) |
| Health expenditure per capita (logged, in 2005 USD) | -0.0008  (0.96) | -0.0140  (0.28) |
| Number of countries | 142 | 144 |
| Country-year observations | 2196 | 2196 |

# Supplementary Appendix SA5: Analyses Using Different Lag Durations

Our default model assumes that any mortality benefits from Global Fund disbursements can first be measured one year after initial disbursement (i.e. lag duration was one year). In this sensitivity analysis, we examine the effect of changing the lag duration from 1 to 4 years. The Table below juxtaposes the main effects for longer lags, next to the default 1-year lag for all-cause adult, all-cause under-5, and malaria-specific under-5 mortality. Global Fund disbursements are strongly associated with all-cause adult mortality reductions within a 1 to 3-year lags. For malaria-specific under-5 mortality, the effect is strongest with a 1-year lag, and the effect diminishes slowly with increasing lag duration. All-cause under-5 mortality remains unrelated to Global Fund disbursements in all analyses, irrespective of the lag assumed.

|  | **Lag duration** | | | |
| --- | --- | --- | --- | --- |
|  | **1 year (default)** | **2 years** | **3 years** | **4 years** |
| Adult mortality (all eligible countries) |  |  |  |  |
| Global Fund $ per capita | **-0·0014  (0**·**005)** | **-0·0018**  **(0·032)** | **-0·0025**  **(0·078)** | -0·0038  (0·145) |
| Global Fund $ per capita$\times$HWD | 0·0013  (0·720) | 0·0002  (0·744) | 0·0004  (0·706) | 0·0011  (0·518) |
| All-cause under-5 mortality (all eligible countries) |  |  |  |  |
| Global Fund $ per capita | -0·0005  (0·330) | -0·0013  (0·124) | -0·0027  (0·062) | -0.0051  (0·059) |
| Global Fund $ per capita$\times$HWD | -0.0005  (0·250) | -0·0005  (0·502) | -0·0005  (0·694) | 0·0001  (0·948) |
| Malaria under-5 mortality |  |  |  |  |
| Global Fund $ per capita | **-0·0069**  **(0**·**033)** | -0·0037  (0·456) | 0·0004  (0·966) | 0·0210  (0·353) |
| Global Fund $ per capita$\times$HWD | 0·0016  (0·400) | 0·0031  (0·296) | 0·0052  (0·333) | 0·0028  (0·836) |

# Supplementary Appendix SA6: Malaria Funding Exposure per Person at Risk

In the default analysis, we analyze the relationship between the under-five malaria-attributed mortality and malaria-targeted disbursements per capita of national population. Another relevant and commonly used standardized measure is malaria funding levels relative to malaria disease burden and need, expressed as funding per person living at risk of malaria, given that the proportion of national populations at risk ranges widely among countries eligible for Global Fund malaria-funding, from 1.67% to 100%.

As a check of robustness, we here repeat our analysis expressing and grouping GF malaria-targeted disbursement levels per person at risk of malaria, using national population-at-risk estimates from the Malaria Atlas Project.

Figure SA6-1 situates GF-eligible countries in terms of their malaria-targeted funding per capita (the default in main paper) and per person at risk (the alternative studied in this sensitivity analysis). As a robustness check, we have repeated the same panel regression as in the right panel of Table 2 of the main paper except using population-at-risk as the malaria disbursement denominator. Despite 17 countries changing funding-level category, the estimated disbursement effects are similar as for the default analysis. A $10 increase in malaria-targeted disbursements per population at risk was associated with a 4.3% reduction in child malaria mortality.

| **Figure SA6-1: Plot of Global Fund-funded countries in terms of their malaria-targeted disbursements cumulatively over 2002-2010, per capita or per person at risk (US $)** |  |
| --- | --- |


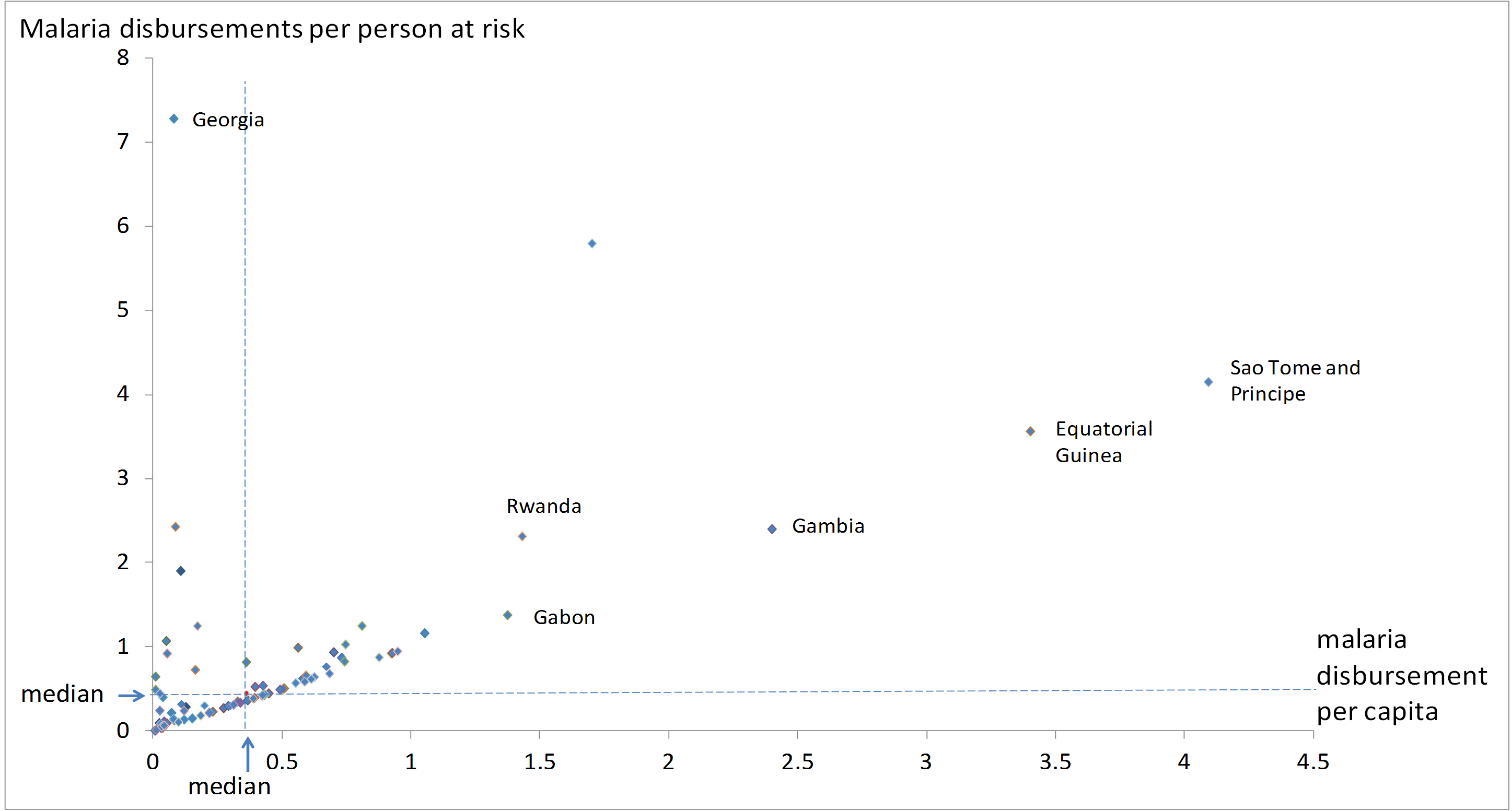


Note:

1. For readability of the graph, we removed Suriname, as an outlier with $1.48 Global Fund disbursement per capita and $25.98 per person at risk. Suriname is still included in the panel regressions shown in Table 2.
2. Average proportion of national population at risk of malaria: 0.73 (range 0.01-1.00) in the highest tertile of recipient countries, 0.74 (range 0.06-1.00) in the middle tertile of countries, and 0.50 in the lowest tertile (range 0.01-0.92).
